# Physical activity in patients with oligo- and polyarticular juvenile idiopathic arthritis diagnosed in the era of biologics: a controlled cross-sectional study

**DOI:** 10.1186/s12969-018-0281-6

**Published:** 2018-10-17

**Authors:** Kristine Risum, Bjørge Herman Hansen, Anne Marit Selvaag, Øyvind Molberg, Hanne Dagfinrud, Helga Sanner

**Affiliations:** 1 Section for Orthopaedic Rehabilitation, Oslo University Hospital, Rikshospitalet, Postboks 4950 Nydalen, 0424 Oslo, Norway; 2 Department of Health Sciences, Institute of Health and Society, Faculty of Medicine, University of Oslo, Oslo, Norway; 3 Department of Sports Medicine, Norwegian School of Sports Sciences, Oslo, Norway; 4 Department of Rheumatology, Oslo University Hospital, Rikshospitalet, Oslo, Norway; 5 Institute of Clinical Medicine, Faculty of Medicine, University of Oslo, Oslo, Norway; 6 National Advisory Unit on Rehabilitation in Rheumatology, Diakonhjemmet Hospital, Oslo, Norway; 7 Norwegian National Advisory Unit on Rheumatic Diseases in Children and Adolescents, Oslo University Hospital, Rikshospitalet, Oslo, Norway; 8 Bjørknes University College, Oslo, Norway

**Keywords:** Juvenile idiopathic arthritis, Physical activity, Sports, Exercise, Facilitators, Barriers, Biologics, Pediatric rheumatology

## Abstract

**Background:**

Knowledge about objectively measured levels of physical activity (PA) and PA participation (included facilitators and barriers for PA) in patients with juvenile idiopathic arthritis (JIA) diagnosed in the era of biologics is limited. We aimed to compare objectively measured PA in patients with oligo- and polyarticular JIA diagnosed in the biologic era with controls and to examine associations between PA and disease variables; furthermore, to explore participation in PA, physical education (PE) and facilitators and barriers for PA participation in patients and controls.

**Methods:**

The study cohort included 60 patients (30 persistent oligo JIA/30 poly-articular disease) and 60 age- and sex-matched controls. Age range was 10–16 years and 83% were female. PA was measured with accelerometry for seven consecutive days. Disease activity, current treatment, disease duration, functional ability, pain and fatigue were assessed. Structured interviews were applied to explore participation in PA and PE, and PA facilitators and barriers.

**Results:**

Patients spent less time in daily vigorous PA than controls, (mean(SE) 21(2) min vs. 26(2) min, *p* = 0.02), while counts per minute (cpm), steps daily, sedentary time and light and moderate PA did not differ. No differences were found between JIA subgroups. The use of biologic medication was associated with higher cpm and lower sedentary time. Most patients and controls participated in organized or unorganized PA and PE, and enjoyment was the most reported facilitator for PA participation. More patients than controls reported pain as a PA barrier.

**Conclusion:**

The PA levels and participation in patients with oligo- and polyarticular JIA are mostly comparable to controls, but patients still need to be encouraged to increase vigorous PA. Enjoyment is the most important facilitator for PA participation in patients with JIA.

## Background

Juvenile idiopathic arthritis (JIA) is the most common pediatric rheumatic disease [1]. The progress in medical therapy has caused a paradigm shift in the management of these patients, reflected by a strong focus on early aggressive treatment, including methotrexate and selective immune-modulators (so-called biological drugs) in recent international guidelines [2]. Accordingly, physiotherapists working with patients with JIA today can focus more on promoting physical activity (PA).

There have been concerns about disease triggering adverse effects of intense PA in JIA, but studies support that exercise is safe [3, 4]. Associations between PA and JIA disease variables are not conclusive. Some studies reported that lower levels of PA were associated with higher disease activity [5, 6], arthritis in weight-bearing joints [6, 7], more pain [8, 9] and lower wellbeing [8], while others did not find any such associations [10, 11].

There is no gold standard method available for measuring PA in children; both *objective* methods including accelerometry, and *subjective* methods like diaries and questionnaires have been applied. Accelerometry is often considered the best option since the method reduces recall bias and social-desirability bias [12]. However, regardless of method used, available studies indicate that patients with JIA have lower levels of PA, spend less minutes in moderate to vigorous PA (MVPA) and more time sedentary than healthy controls despite advances in the multidisciplinary management of JIA [6–8, 10, 11, 13, 14].

The World Health Organization (WHO) recommends children with and without disabilities to do a minimum of 60 min of MVPA daily [15]. Previous studies indicate that patients with JIA meet these recommendations less frequently than healthy controls [6–8, 10]. Vigorous PA (VPA) is considered more beneficial for health outcomes than moderate PA (MPA) [16, 17]. Knowledge is sparse on objectively measured PA levels and intensities in patients with JIA diagnosed in the era of biologics and whether their PA behavior is optimal to gain health benefits. Furthermore, these patients seem to participate more in unorganized than in organized PA [18, 19], but PA facilitators and barriers need to be identified [20]. Also, little is known about participation in physical education (PE) in school. Increased knowledge about PA participation is needed to help health professionals promote a physically active lifestyle for patients with JIA.

Thus, the objectives of this cross-sectional study were to 1) compare objectively measured levels and intensities of PA between JIA subgroups (oligo- and polyarticular) diagnosed in the era of biologics and an age- and sex-matched control population; 2) to assess differences in PA between JIA subgroups and examine associations between PA and disease variables and 3) to explore participation in PA and PE and facilitators and barriers for PA in the patients and the matched controls.

## Methods

### Study participants

The inclusion criteria for patients were: (A) age 10–16 years, (B) disease duration > 6 months (to ensure that patients had started anti-inflammatory medication if needed), (C) JIA classified as persistent oligoarthritis or polyarticular disease (extended oligoarthritis and polyarticular RF +/−) according to the International League of Associations for Rheumatology (ILAR) criteria [21], and (D) home address in the geographical area served by the South-Eastern Norway Regional Health Authority. This area has a denominator population of 2.8 million (57% of the Norwegian population).

Patients were excluded if they had comorbidities associated or potentially associated with, impaired cardiopulmonary fitness (e.g heart- or lung disease), severe orthopedic conditions, recent surgery or inability to walk. These exclusion criteria were applied because the patients were also included in a parallel study with compulsory exercise tests.

We consecutively recruited eligible patients with a planned routine visit at Oslo University Hospital (OUS), during 2015 until the predefined number of 30 in each subgroup was reached.

Individually age- and sex-matched controls from the general population (living in or nearby Oslo) were randomly selected from the National Registry (a registry of all individuals living in Norway), and were invited to participate by mail. Exclusion criteria for the controls were inflammatory rheumatic or autoimmune disease, severe heart or lung disease, or other diseases involving mobility problems.

All participants provided written informed consent/assent. The study was approved by the Norwegian South East Regional Ethics Committee for Medical Research (2014/188).

### Data collection and clinical examination

All patients were clinically examined in conjunction with their routine visit at OUS between January and August 2015. All controls were examined during a one-day program between November 2015 and March 2016 at OUS. Height and bodyweight were measured to the nearest 0.1 cm and 0.1 kg, respectively, with participants wearing light clothes and no shoes. Body mass index (BMI) was calculated and the age- and sex-specific BMI cut-off values were used to categorize the children as normal weight, overweight or obese [22] . Pain and fatigue were assessed with the following questions: “How do you rate your pain/fatigue in the previous week?” and “How do you rate your current pain?” We used the numeric rating scale (NRS) 0–10, where 0 = no pain/fatigue and 10 = worst possible pain/fatigue [23]. ESR and CRP were analyzed according to hospital routine.

### Objectively measured physical activity

Volume and intensity of PA were measured using Actigraph GT3X+ accelerometers (ActiGraph, Pesacola, FL, USA), which measures bodily acceleration. Participants were instructed to wear the accelerometer for seven consecutive days during waking hours, except during swimming, bathing, and other water activities since the device is not waterproof. The accelerometer was worn on an elastic belt at the waistline on the right side of the hip. The participants noted time spent on swimming, cycling and skiing, as the accelerometer does not capture these physical activities accurately. Movement is detected as a combined function of the frequency and intensity of movement. Vertical axis count data were exported from the device in 10-s epochs using the ActiLife 6 software (ActiGraph, Pesacola, FL, USA). The raw data were converted to mean *counts per minutes* (cpm) (our main outcome) and mean *steps per day* to reflect the general level of PA. We applied the most used cut-off points regarding PA intensities in children; sedentary time (< 101 cpm), light PA (LPA) (≥101 to ≤2295 cpm), moderate PA (MPA) (≥2296 to ≤4011 cpm) and vigorous PA (VPA) (> 4011 cpm) [24]. Non-wear periods were defined as consecutive strings of zero counts lasting at least 10 min. In order for a day to be deemed valid, participants had to accumulate at least 8 h of valid wear. Only participants who had worn the accelerometer for at least 3 days were included in the analyses.

### Subjectively measured physical activity

To explore participation in PA and PE, and facilitators and barriers for PA participation, a senior physiotherapist (KR) performed a structured 15–20 min interview with all participants individually. The participants could choose if they wanted parent(s) to be present during the interview. The interview guide was developed for this study by two physiotherapists and one nurse (all experienced in pediatric rheumatology), based on literature review and clinical experience. The questions included were: 1) Do you participate in any organized and/or unorganized physical activity? If yes, which activity/activities? 2) Do you perceive barriers to being physical active? If yes, how? 3) Do you perceive facilitating factors to being physical active? If yes, which? and 4) Do you participate in physical education classes in school? If yes, how often? If the participants replied positively to the initial question, follow-up questions were asked. If needed, the interviewer provided some examples during the follow-up questions. The responses were written down during the interviews.

### Assessment of disease variables in patients

Disease activity was assessed by the Juvenile Arthritis Disease Activity Score 71 (JADAS 71) [25]. The children’s score of the patients/(parents) global assessment was used to calculate the JADAS 71 score. The joint assessments were performed by a senior physical therapist (KR). Clinical inactive disease (CID) was defined according to the Wallace criteria [26]. Disease duration and medication history were obtained from the patients’ medical records. The Childhood Health Assessment Questionnaire (CHAQ) was used to measure functional ability [27, 28]. The children completed the CHAQ, with assistance from their parents if needed.

### Statistical analysis

Continuous data were expressed as mean (standard deviation (SD) or median (25th–75th percentile) as appropriate and categorical data as n (%). Independent sample t tests, analyses of covariance, Mann Whitney U tests or chi-square tests were used to assess differences between patients and controls and between patient subgroups as appropriate. Linear regression analyses were used to identify correlates of cpm, vigorous PA and sedentary time in patients. Disease related variables that were associated (*p* < 0.15) with the outcome variables in univariate analyses, were evaluated in the multivariate analyses (method enter), adjusted for age, sex, and accelerometer wear time. To be able to perform frequency analyses, variables for PA and PE participation, facilitators and barriers were categorized and coded as reported (1) and not reported (0) according to the participant’s responses. Statistical tests were conducted using SPSS version 23.0 (SPSS, Chicago, Illinois, USA). *P* values < 0.05 were considered statistically significant. Due to multiple statistical analyses, *p*-values close to 0.05 should be interpreted with caution. Effect size for difference in PA categories was determined by using the partial Eta Squared value, and were defined as small = 0.2, medium = 0.5 or large = 0.8.

## Results

### Study participants

**Fig. 1:**
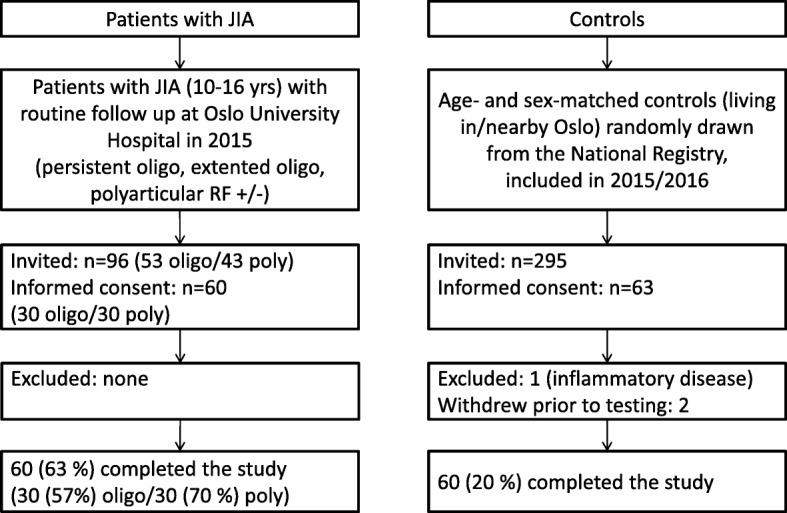
Flowchart over participant inclusion. *JIA* juvenile idiopathic arthritis, *RF* rheumatoid factor

Of all patients who were invited to participate, 60/96 (63%) accepted (Fig. 1); this included 10/22 (45%) of the invited boys and 50/74 (68%) of the invited girls. The JIA patient cohort consisted of 60 consecutive patients, 30 with oligoarthritis and 30 with poly JIA. In the poly JIA group, 15 patients had poly JIA from disease onset (14 of these were RF÷, and one was RF+) and 15 had an extended oligo JIA (Fig. 1).

### Health related measures in patients and controls

Measures of height, weight and BMI did not differ between patients and controls (Table 1). Two patients and six controls were categorized as overweight, while two patients and two controls were categorized as obese. Patients and controls reported comparable levels of current pain and similar levels of pain and fatigue during the previous week. Only three patients and none of the controls had CRP > 4 mg/l, whereas all patients and controls had normal range ESR values.

**Table 1:** Characteristics of patients with JIA and controls

	Oligo JIA (*n* = 30)	Poly JIA (*n* = 30)	*P*-value oligo vs poly JIA	JIA total (*n* = 60)	Controls (*n* = 60)	*P*-value JIA total vs controls
Age (yrs)	13.5 (2.2)	13.7 (2.2)	0.62	13.6 (2.2)	13.5 (2.6)	0.86
Female sex, n (%)	27 (90)	23 (77)	0.17	50 (83)	50 (83)	1.00
Height (cm)	157.1 (11.8)	158.7 (13.6)	0.64	157.9 (12.6)	161.2 (12.6)	0.16
Weight (kg)	47.0 (10.1)	51.5 (16.2)	0.21	49.3 (13.8)	53.5 (15.4)	0.12
BMI (kg/m^2^)	18.8 (2.1)	20.1 (4.4)	0.17	19.4 (3.5)	20.2 (3.5)	0.26
NRS current pain (0–10), n (%) with score > 0	12 (40)	11 (37)	0.76	23 (38)	18 (30)	0.34
NRS pain previous week (0–10)	0.0 (2.0–3.3)	1.0 (0.0–3.5)	0.58	1.0 (0.0–3.0)	1.0 (0.0–3.0)	0.67
NRS fatigue previous week (0–10)	3.5 (2.0–6.3)	3.0 (2.0–5.3)	0.61	3.0 (2.0–6.0)	3.0 (1.0–3.5)	0.09
CRP > 4 mg/l, n (%)	1 (3)	2 (7)	1.00	3 (5)	0 (0)	0.24
Physiotherapy regularly, n (%)	3 (10)	10 (33)	0.03	13 (22)	4 (7)	0.04
Disease duration (yrs)	7.6 (3.9)	7.3 (4.0)	0.74	7.5 (3.8)	NA	
JADAS 71 (0–101)	3.3 (0.8–4.8)	3.2 (1.4–4.6)	0.80	3.3 (1.1–4.8)	NA	
CHAQ (0–3)	0.1 (0.0–0.3)	0.0 (0.0–0.4)	0.62	0.0 (0–1.4)	NA	
Off medication, n (%)	10 (33)	2 (7)	0.02	12 (20)	NA	
NSAIDs regularly, n (%)	9 (30)	7 (23)	0.56	16 (27)	NA	
Synthetic DMARDs, n (%)	18 (60)	22 (73)	0.27	40 (67)	NA	
MTX, n (%)	17 (57)	21 (70)	0.28	38 (63)	NA	
Sulfasalazine, n (%)	1 (3)	1 (3)	1.00	2 (3)	NA	
Biologic DMARDs, n (%)	5 (17)	20 (67)	< 0.001	25 (42)	NA	
TNFi, n (%)	5 (17)	18 (60)	0.001	23 (38)	NA	
IL-6i, n (%)	0 (0)	2 (7)	0.49	2 (3)	NA	
Synthetic+biologic DMARDs, n (%)	5 (17)	14 (47)	0.01	19 (32)	NA	
Active disease, n (%)	18 (60)	22 (73)	0.27	40 (67)	NA	
Clinical inactive disease, n (%)	12 (40)	8 (27)		20 (33)	NA	

Numbers are mean (SD) or median (25th -75th percentile) unless otherwise indicated

*JIA* juvenile idiopathic arthritis, *Oligo JIA* persistent oligoarticular JIA, *Poly JIA* extended oligoarticular JIA and polyarticular JIA RF +/−, *JIA total* persistent oligoarticular JIA, extended oligoarticular JIA and polyarticular JIA RF +/−, *RF* rheumatoid factor, *BMI* body mass index, *NRS* numeric rating scale, *CRP* C-reactive protein, *JADAS* juvenile arthritis disease activity score, *CHAQ* childhood health assessment questionnaire, *NSAIDs* non-steroid anti-inflammatory drugs, *DMARDs* disease modifying anti-rheumatoid drugs, *MTX* methotrexate, *TNFi* tumor necrosis factor inhibitors, *IL-6i* interleukin-6 inhibitor, *NA* not applicable

### Disease characteristics and treatment in the JIA cohort

The patients in both JIA subgroups had relatively modest disease activity (Table 1), and their functional limitations were in the range of no to mild measured by CHAQ [29]. Fifteen (25%) of the patients had active joint disease (range one-two joints), with affliction of lower extremities in nine patients, the upper extremities in five, the neck in one, and the temporomandibular joint in one. Current treatment is shown in Table 1. Twenty-five (42%) of patients used biologic DMARDs, most commonly TNFi; of the 35 (58%) patients not on biologics, 25 had oligo- and 10 polyarticular JIA. None of the patients were on corticosteroids.

### Objectively measured physical activity

One patient did not return the accelerometer and four patients and four controls had less than three valid wear days and were therefore excluded from the analyses. Thus, acceptable data from the accelerometers were retrieved in 55 patients (47 girls and eight boys) and 56 controls (47 girls and nine boys) (Table 2). We found no differences between patients and controls regarding cpm, steps daily, sedentary time, LPA, MPA or proportion achieving the WHO recommendations for MVPA. However, the patients spent less time in daily VPA than the controls, mean (SE) 21 (2) min vs 26 (2) min respectively, *p* = 0.02. The effect size was small (partial Eta Squared = 0.05). Adjusting the analyses for wear month did not alter any findings. Self-reported time spent on swimming, bicycling and skiing were generally of short duration and were not significantly different between patients and controls, and therefore not included in the analyses.

**Table 2:** Physical activity measured by accelerometers in patients with JIA and controls

Accelerometer variables	Oligo JIA (*n* = 28)	Poly JIA (*n* = 27)	*P*-value oligo vs poly JIA	JIA total (*n* = 55)	Controls (*n* = 56)	*P*-value JIA total vs controls
Counts per minute ^a^	437 (140)	478 (233)	0.43	457 (91)	479 (132)	0.48
Steps daily ^a^	8932 (2307)	9563 (2951)	0.38	9242 (2637)	9694 (2572)	0.36
Sedentary PA daily (min) ^b^	580 (11)	573 (11)	0.63	577 (7)	573 (7)	0.86
Light PA daily (min) ^b^	186 (9)	192 (9)	0.65	190 (6)	182 (6)	0.38
Moderate PA daily (min) ^b^	32 (2)	34 (2)	0.44	33 (2)	36 (2)	0.09
Vigorous PA daily (min) ^b^	21 (2)	20 (2)	0.88	21 (2)	26 (2)	0.02
Achieves 60 min MVPA daily, n (%)	10 (36)	8 (30)	0.63	18 (33)	27 (48)	0.10
Accelerometer wear time (min) ^a^	812 (60)	827 (34)	0.25	819 (49)	816 (46)	0.71

*JIA* juvenile idiopathic arthritis, *Oligo JIA* persistent oligoarticular JIA, *Poly JIA* extended oligoarticular JIA and polyarticular JIA RF +/−, *JIA total* persistent oligoarticular JIA, extended oligoarticular JIA and polyarticular JIA RF +/−, *RF* rheumatoid factor, *PA* physical activity, *MVPA* moderate-to-vigorous physical activity

^a^Mean (SD). ^b^ Mean (SE) adjusted for accelerometer wear time

No significant differences in accelerometer variables were found between the included JIA subsets (Table 2). Thus, the regression analyses were conducted for the JIA sample as one group. Also, no significant difference was found between patients with CID and controls for cpm; mean (SD) 465 (215) vs 479 (132), *p* = 0.74.

### Correlates of physical activity in patients with JIA

For cpm, use of biological medication and participation in organized PA were identified as correlates, in addition to lower age (Table 3). For VPA, only participation in organized PA was identified as a correlate. For lower sedentary time, lower age and using biological medicine were significant correlates, in addition to accelerometer wear time. Disease variables that were not associated with the outcome variables in univariate analyses (*p* > 0.15) included: use of any medication, use of methotrexate, CRP, ESR, having active joints, having active joints in the lower extremities, JADAS 71, CHAQ, disease duration, current pain, and pain and fatigue during the previous week.

**Table 3:** Correlates for physical activity in patients with JIA (*N* = 55)

	Univariate Analyses		Multiple Regression Analyses	
	Unstandardized B (95% CI)	*P*-value	Unstandardized B (95% CI)	*P*-value
Counts per minute ^a^				
Age	−31.5 (− 54.1, − 8.9)	0.007	−25.2 (−46.0, − 4.4)	0.02
Female sex	100.2 (− 44.7, 245.2)	0.17	20.4 (111.7, 152.4)	0.76
Participation in organized PA	141.0 (38.7, 243.2)	0.008	105.7 (9.2, 202.2)	0.03
Use of biologic medication	150.5 (53.5, 247.5)	0.003	117.5 (24.2, 210,7)	0.02
Disease duration	−9.5 (− 22.7, 3.7)	0.15		
R^2^ adjusted			0.37	
Vigorous physical activity ^b^				
Age	0.4 (−1.0, 1.9)	0.57	0.5 (−0.9, 1.9)	0.41
Female sex	7.2 (−0.9, 16.1)	0.08	5.69 (−2.8, 14.2)	0.19
Participation in organized PA	8.4 (2.3, 14.5)	0.008	7.6 (1.3, 13.9)	0.02
Use of biologic medication	5.0 (−1.1, 11.2)	0.10		
Accelerometer wear time	0.03 (−0.04, 0.09)	0.41	0.03 (−0.04, 0.09)	0.41
R^2^ adjusted			0.11	
Sedentary time ^b^				
Age	21.2 (14.7, 27.6)	< 0.001	17.4 (12.1, 22.6)	< 0.001
Female sex	−33.4 (18.6, −85.5)	0.20	−8.9 (−41.0, 23.3)	0.32
Participation in organized PA	−30.2 (−68.5, 8.1)	0.12		
Use of biologic medication	−43.8 (−79.6, −8.0)	0.02	−31.0 (−53.9, − 8.0)	0.01
Arthritis in lower extremities	46.3 (−2.4, 95.1)	0.06		
Disease duration	4.0 (−0.7, 8.7)	0.09		
Accelerometer wear time	0.8 (0.4, 1.1)	< 0.001	0.6 (0.4, 0.8)	< 0.001
R^2^ adjusted			0.65	

*JIA* juvenile idiopathic arthritis, *CI* confidence interval, *PA* physical activity

^a^Results from the final model of multiple linear regression analysis (method enter) controlled for age and sex. ^b^ Results from the final models of multiple linear regression analyses (method enter) controlled for age, sex and accelerometer wear time

### Participation in physical activities and physical education

Participation in organized and unorganized PA were not significantly different between patients and controls (Table 4). The most commonly practiced organized and unorganized modes of PA are shown in Table 4. Nearly all the patients (58 (97%)) and the controls (59 (98%)) reported that they participated regularly in PE (Table 4). However, 25% of the patients reported that they occasionally needed some modification of the activities in PE at school.

**Table 4:** Participation in physical activity and physical education in patients with JIA and controls

	Patients with JIA (*n* = 60)	Controls (*n* = 60)	*p*-value
Participation in PA (organized and/or unorganized)	51 (85)	56 (93)	0.14
Participation in organized PA	38 (63)	47 (78)	0.11
Frequency of organized PA			0.14
None	22 (37)	13 (22)	
1–3 h/week	16 (27)	12 (20)	
4–6 h/week	12 (20)	24 (40)	
7–9 h/week	8 (13)	6 (10)	
> 10 h/week	2 (3)	5 (8)	
The most reported organized PA			
Dancing	14 (23)	9 (15)	0.25
Soccer	10 (17)	16 (27)	0.18
Handball	5 (8)	5 (8)	1.00
Cross-country skiing/biathlon	4 (7)	5 (8)	0.73
Swimming	3 (5)	4 (7)	0.70
Horse riding	4 (7)	2 (3)	0.34
Athletics	1 (2)	4 (7)	0.36
Fight sports (taekwondo, kickboxing, boxing)	0 (0)	5 (8)	0.06
Participation in unorganized PA	41 (68)	42 (70)	1.00
Frequency of unorganized PA			0.79
None	19 (32)	18 (30)	
1–3 h/week	30 (50)	34 (57)	
4–6 h/week	11 (18)	8 (13)	
The most reported unorganized PA			
Jogging/running	10 (17)	14 (23)	0.36
Training in fitness center	11 (18)	10 17)	0.81
Strength exercising at home	10 (17)	8 (13)	0.61
Walking/hiking	5 (8)	10 (17)	0.17
Ball activities	3 (5)	4 (7)	0.70
Cross-country skiing	4 (7)	2 (3)	0.68
Swimming	3 (5)	1 (2)	0.62
Participation in PE			< 0.001
Always (without modifications)	42 (70)	59 (98)	
Always (occasionally with modifications)	16 (27)	0 (0)	
Sometimes	2 (3)	1 (2)	

Numbers are n (%)

*JIA* juvenile idiopathic arthritis, *PA* physical activity, *PE* physical education

### Facilitators and barriers for physical activity

Barriers for participating in PA were reported by 26 (43%) patients and 19 (32%) controls. The most reported barrier was pain in patients and time in controls (Table 5). The most frequently reported facilitators for PA in both groups were enjoyment and becoming fit.

**Table 5:** Facilitators and barriers for being physically active in patients with JIA and controls

	Patients with JIA (*n* = 60)	Controls (*n* = 60)	*P*-value
Facilitators for being physically active			
Enjoyment	40 (67)	45 (75)	0.32
Become/stay fit	12 (20)	21 (35)	0.07
Social setting/be with friends	1 (2)	13 (22)	0.001
Less pain	4 (7)	0 (0)	0.12
Barriers for being physically active			
Pain	18 (30)	8 (13)	0.03
Time	3 (5)	11 (18)	0.04
Disease activity	4 (7)	0 (0)	0.12
Lack of energy	2 (3)	2 (3)	1.00

Numbers are n (%)

*JIA* juvenile idiopathic arthritis, *PA* physical activity

## Discussion

The main finding of our study was that the general level of PA in patients with JIA was comparable with age- and sex-matched controls, but patients spent less time in vigorous PA. The use of biologics was associated with higher levels of PA. Also, patients engaged in similar physical activities as controls, almost all participated in PE, and enjoyment was the most frequently reported facilitator. To our knowledge, this is the first study to a) directly compare PA and PE in patients with JIA diagnosed in the biologic era with matched controls examined in the same time period and b) comprehensively measure PA objectively, and assess correlates, facilitators and barriers for PA in the same study population.

Regarding representativeness of our patients, the included JIA categories constitute 75% of patients with JIA included in our hospital-based registry; thus, the results cannot be extrapolated to the categories not included. However, a previous study found no differences across all ILAR categories when assessing PA by accelerometry [7]. The proportion of girls in our cohort was slightly higher compared to other studies on PA in JIA [6, 7]. We believe the reason for this is twofold; most ILAR categories which were not included have a less female predominance than included categories and the study participation rate was higher among eligible girls than boys. We cannot rule out that the patients enrolled might be biased towards more physically active patients with a milder disease than those who declined participation. However, we are not allowed to report data on patients declining to participate.

The controls were randomly selected from the National Registry, and were examined within a year after the patients, thereby avoiding bias due to changes in patterns of PA. The levels of PA and PA participation in our controls were comparable to recent, population-based studies of Norwegian children [30, 31], indicating that the controls were representative.

We found that most objectively measured PA parameters, including overall cpm, MPA, LPA, sedentary time and proportion achieving the WHO recommendations for MVPA were not significantly different in patients and controls. These findings are in contrast to other studies reporting that patients with JIA have lower cpm [6, 7, 10], and spend less time in MPA and LPA [6, 7] and more in sedentary time [10] than controls. However, in most of these studies, included patients were diagnosed both before and after the introduction of biological medications. We applied the most widely used PA intensity thresholds [24]; in lack of international consensus it is challenging to directly compare our data with PA intensity data from other studies. Adjusting our analyses for wear month did not alter our results, indicating that seasonality did not have a major impact on PA.

Similar to other studies, the time devoted to VPA was lower in our patients than in controls [6, 7, 10]. Even if the effect size for the difference was small, it may be of clinical importance when aiming to optimize the health benefits of PA. Patients with JIA have increased risk for early subclinical atherosclerosis [32]. VPA is particularly important to reduce the risk of cardiovascular diseases [16, 17]. Therefore, patients with JIA should be recommended to include VPA in their PA behavior, but until now, we have not provided specific advice on VPA. Since our patients spent nearly 10 h in daily sedentary time, it seems reasonable to also focus on limiting sedentary behavior to reduce the risk of cardiovascular diseases.

Our identified correlates of objectively measured PA in patients were mostly in line with studies in healthy children. Lower age was associated with higher cpm and lower sedentary time [33], and participation in organized PA was associated with higher cpm and VPA [31, 34]. In healthy children, boys have higher PA levels than girls [33]. We found no association with sex, which must be interpreted with caution due to a low proportion of boys. Interestingly, the use of biological medication was associated with higher cpm and lower sedentary time. This may reflect the effectiveness of these medications, but also that patients using biologics have regular contact with health professionals who repeatedly encourage them to be physically active. Other disease related variables were not identified as correlates; this included also pain which is in accordance with other studies [5–7, 10] and fatigue, which is contrary to another study [35]. Interestingly, our patients and controls reported similar low levels of pain and fatigue.

Participation in organized and unorganized PA were not significantly different between patients and controls. A higher proportion of our patients participated in organized PA than previously reported [19], which may be favorable because of its association with higher cpm and VPA. Also, we found higher PE participation compared to recent studies [8, 35, 36]. However, PE participation has been categorized differently in previous studies, making comparisons difficult. The types of physical activities our patients reported are comparable to activities reported in a national sample of healthy Norwegian children and adolescents [31].

Enjoyment was the most frequently reported facilitator for PA participation in both patients and controls who were regularly physically active, followed by becoming fit. The importance of enjoyment for PA participation has also previously been highlighted in patients with JIA [37] and healthy children [33]. Having less pain was a facilitator in some of our patients, supporting existing results [37]. Both patients and controls reported barriers for PA participation. More patients reported pain, while more controls reported time as a barrier, and none of the study participants reported fatigue as a PA barrier. Disease activity was a barrier in only a few patients (7%). Taken together, disease related barriers (i.e. pain and disease activity) were more common than regular barriers (i.e. time) in patients, similar to findings in other studies [37, 38].

We believe the main reasons for our positive results are two-fold: Firstly, the health care system in Norway has from year 2000 allowed for relatively early introduction of biologics, securing that the patients are aggressively treated following international recommendations [2]. All patients were diagnosed after 2000 and 42% was currently treated with biologics. They seem well treated, supported by measures of modest disease activity, low functional disability and low inflammatory parameters. Interestingly, a recent study measuring PA levels with a questionnaire reported comparable overall PA levels between patients with JIA (with low disease activity treated with a treat-to-target approach) and controls [39]. Secondly, the physiotherapy management of all patients newly diagnosed with JIA at OUS includes individualized tailored patient education regarding the importance and safety of PA. They have from 2003 been encouraged to participate in PA and PE like their healthy peers without any general restrictions (even if they have active arthritis). Specific exercise programs are not used anymore because patients have improved functional ability and our experience is that there is poor adherence to such programs, which is in line with previous research [40, 41]. To facilitate PA and PE participation, there is also a close collaboration between health professionals at OUS, local physiotherapists, PE teachers and patients and parents.

The cross-sectional design does not allow for the assessment of the causal relation between study outcomes and explanatory factors. Also, measuring a complex behavior like PA at one time point may not provide a complete picture of an individual’s PA behavior. Furthermore, to our knowledge, disease-specific facilitators and barriers are not addressed in standardized questionnaires. Therefore, we used a structured interview to assess these factors and PA participation, which may have limited the generalizability of the results. Another limitation is that no formal power analyses were performed for the outcomes; we have a relatively small sample size, which might have introduced type 2 errors.

## Conclusions

Even though most PA levels and PA participation were comparable between older children and adolescents with oligo- and polyarticular JIA diagnosed in the biologic era and controls, patients spent less time in VPA. Health professionals should take the patient’s preferences about enjoyable activities and disease symptoms like pain into account when encouraging a physically active lifestyle, including more VPA to optimize the health benefits of PA.

## Abbreviations

BMI: Body mass index
CHAQ: Childhood health assessment questionnaire
Cpm: Counts per minute
CRP: C-reactive protein
DMARDs: Disease-modifying anti-rheumatic drugs
ESR: Erythrocyte sedimentation rate
IL-6i: Interleukin-6 inhibitor
ILAR: International League of Associations for Rheumatology
JADAS: Juvenile arthritis disease activity score
JIA: Juvenile idiopathic arthritis
LPA: Light physical activity
MPA: Moderate physical activity
MVPA: Moderate-to-vigorous physical activity
NRS: Numeric rating scale
NSAIDs: Non-steroid anti-inflammatory drugs
OUS: Oslo University Hospital
PA: Physical activity
PE: Physical education
RF: Rheumatoid factor
TNFi: Tumor necrosis factor inhibitors
VPA: Vigorous physical activity
WHO: World Health Organization
